# Engineered Janus hydrogels: biomimetic surface engineering and biomedical applications

**DOI:** 10.1093/nsr/nwae316

**Published:** 2024-09-09

**Authors:** Mingfei Pan, Tao Shui, Ziqian Zhao, Li Xiang, Bin Yan, Ning Gu, Hongbo Zeng

**Affiliations:** Department of Chemical and Materials Engineering, University of Alberta, Alberta T6G 1H9, Canada; Changzhou Second People's Hospital, Changzhou Medical Center, Nanjing Medical University, Changzhou 213164, China; School of Materials Science and Engineering, Southeast University, Nanjing 211189, China; Department of Chemical and Materials Engineering, University of Alberta, Alberta T6G 1H9, Canada; School of Mechanical Engineering, Jiangsu Key Laboratory for Design and Manufacture of Micro-Nano Biomedical Instruments, Southeast University, Nanjing 211189, China; National Engineering Laboratory for Clean Technology of Leather Manufacture, College of Biomass Science and Engineering, Sichuan University, Chengdu 610065, China; Nanjing Key Laboratory for Cardiovascular Information and Health Engineering Medicine, Institute of Clinical Medicine, Nanjing Drum Tower Hospital, Medical School, Nanjing University, Nanjing 210093, China; Department of Chemical and Materials Engineering, University of Alberta, Alberta T6G 1H9, Canada

**Keywords:** interfacial science, bionics, hydrogel bioadhesives, asymmetric surfaces

## Abstract

Hydrogel bioadhesives, when applied to dysfunctional tissues substituting the epidermis or endothelium, exhibit compelling characteristics that enable revolutionary diagnostic and therapeutic procedures. Despite their demonstrated efficacy, these hydrogels as soft implants are still limited by improper symmetric surface functions, leading to postoperative complications and disorders. Janus hydrogel bioadhesives with unique asymmetric surface designs have thus been proposed as a reliable and biocompatible hydrogel interface, mimicking the structural characteristics of natural biological barriers. In this comprehensive review, we provide guidelines for the rational design of Janus hydrogel bioadhesives, covering methods for hydrogel surface chemistry and microstructure engineering. The engineering of Janus hydrogels is highlighted, specifically in tuning the basal surface to facilitate instant and robust hydrogel-tissue integration and modulating the apical surface as the anti-adhesion, anti-fouling, and anti-wear barrier. These asymmetric designs hold great potential in clinical translation, supporting applications including hemostasis/tissue sealing, chronic wound management, and regenerative medicine. By shedding light on the potential of Janus hydrogels as bioactive interfaces, this review paper aims to inspire further research and overcome current obstacles for advancing soft matter in next-generation healthcare.

## Teaser

This review provides guidelines for the rational design of Janus hydrogel bioadhesives, covering methods for hydrogel surface chemistry/microstructure engineering and asymmetric basal/apical surface designs for clinical translation.

## INTRODUCTION

Recent advances in biomedical soft matter have offered a good opportunity to design various types of diagnosis and treatment processes with minimal invasion. Although extensive progress has been made in the field of biomaterials such as bioadhesives, biosensors, and bioelectronics, interventional procedures are still limited by many bottlenecks such as poor biocompatibility, mechanical mismatch with the surrounding tissues, and unexpected microbial accumulation in complex biological environments [1–5].

Hydrogels are three-dimensional (3D) polymeric networks that retain a large amount of water. Due to their physically and chemically crosslinked networks, hydrogels exhibit similar mechanical properties with soft tissues being the combination of viscous fluidity and elastic solidity (viscoelastic behavior) [4,6,7]. Meanwhile, the mobile ions and electrons in the hydrogels make them ideal candidates as the conductive soft platforms coupling with other electronics to assemble hydrogel-based electronics [8–10]. To this end, a conformal, continuous, and stable interface is expected by attaching hydrogel onto biological tissues, benefiting a wide range of biomedical operations. The expanding demands of human-machine interfaces have also promoted the field of hydrogel-based hybrid systems in the past decade. For instance, hydrogel-based artificial skins [11–16] with sensing functions of pressure, deformation, temperature, and humidity have been designed and utilized for *ex vivo* applications of healthcare. However, most of the existing hydrogels lack the proper surface functions to fulfill the requirement of *in vivo* biomedical procedures [17–19]. There are two major challenges that prevent adhesive hydrogels from replacing conventional fixation materials such as staples, sutures, and cyan-based glues in clinical procedures: (i) first of all, the adhesives should first penetrate the hydration layer on the surfaces of target tissues and organs prior to the process of adhesion formation, resulting in a long curing time and insufficient adhesion strength for surgery operation; (ii) on the other hand, undesirable adhesion to surrounding tissues always occurred at the opposite side of the adhesive hydrogel, known as postoperative adhesion complications in medicine. Moreover, hydrogels applied onto the human organ generates a new surface that replaces the original epithelium. The dissimilarity of the newly generated hydrogel surface to the original epithelium can lead to a series of postoperative complications and dysfunctions [20–22]. Janus hydrogels, named after the two-faced Roman god Janus, are a kind of novel material with distinct properties on opposing sides [23]. These unique hydrogels with an apical-basal asymmetric surface characteristic have attracted tremendous attention recently due to their immense potential for clinical translation. In practical biomedical scenarios, robust wet adhesion is regularly required at the basal side of the adhesive hydrogels facing the tissue epitheliums while the hydrogel apical surface properties should be similar to the original epitheliums [21,24–26]. Although the advantages of Janus hydrogels as bioadhesive interfaces are gradually recognized, the fundamental understanding of hydrogel surface characteristics is restricted. It is of the utmost importance to identify the critical surface chemistry and geometry of hydrogels that influence their biomedical functions for their utilization as artificial soft implants and regenerative medicine [27–30].

**Figure 1.**
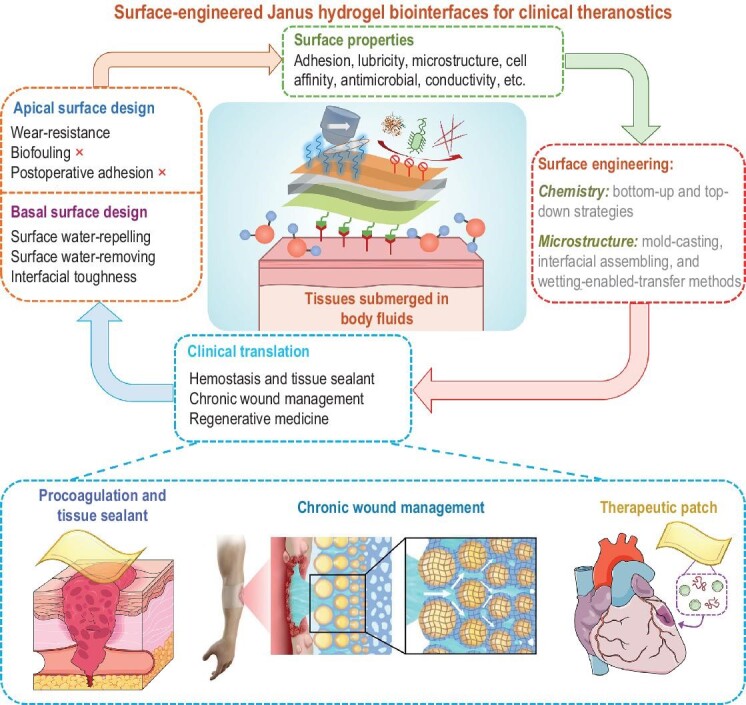
Design and development of Janus hydrogels as bioadhesive interfaces with strategies for engineering asymmetric surface functions and their potential clinical applications.

Whereas hydrogel-based biomaterials have already made a tremendous impact on biomedical research and clinical practice, the significance of surface design on hydrogels has been undervalued. In this review, we systematically discuss the mechanisms of diverse surface chemistry and microstructure on hydrogels and their biological effect on different tissues which is still absent in the field (Fig. 1). Meanwhile, we also summarize the functional layer structure design for hydrogels targeting the terminal requirements from clinical operations which can efficiently support modern healthcare applications in various biomedical fields, including hemostasis/tissue sealing, chronic wound management, and regenerative medicine.

## BIOINSPIRED HYDROGEL SURFACE ENGINEERING

The biological effect of an exogenous material commonly originates from two aspects: (i) the mechanical response generated by the bulk properties of the material; (ii) tissue-material interactions based on surface properties of the material. As a porous three-dimensional soft network, hydrogels actively interact with the surrounding environment in the biological system which makes their surface properties extremely important, being responsible for the corresponding biological responses. In this section, the effect of surface adhesion, lubrication, geometry, cell affinity, electrical, and antimicrobial properties on biological response is first discussed. The methods for surface engineering that help alter the hydrogel surface properties are thus systematically summarized.

### Hydrogel surface properties

#### Adhesion

Considering the fact that most natural tissues and synthetic hydrogels contain water as the majority constituent, strong adhesion could never be achieved with water molecules due to their weak bonding energy [27]. At the hydrogel-tissue interfaces, the adhesion greatly relies on their minor constituents (e.g. polymers and proteins) and requires a specific design to form stable interfacial bonds for robust adhesion performance [31–33]. The interfacial bonds can be classified as non-covalent and covalent bonds. Covalent bonds between hydrogels and tissues depend on reactive functional groups at tissue surfaces including thiol groups, hydroxyl groups, amine groups, and carboxyl groups. For instance, hydroxyl groups of the tissue surfaces can react with borate acid to form borate ester bonds, amine groups can react with carboxyl and aldehyde groups to form amide and imine bonds, respectively [34–36]. Covalent bonds offer strong binding energy (200 to 1000 kJ/mol) for stable adhesion but require long curing time (minutes to hours) or external assistance (e.g. ultraviolet light, heat, pH, and catalysts) to approach appreciable yet reversible adhesion [37]. Non-covalent bonds for bio-adhesion typically refer to hydrogen bonds, electrical double layer interactions (in physiological saline), hydrophobic interactions, and π-π interactions [38–41]. When compared with covalent bonds, non-covalent bonds offer relatively low binding energy and are sensitive to the environment (pH, temperature, magnetic field, etc.), leading to reversible adhesion. However, the deployment process of non-covalent interactions is rapid which is desirable for instant adhesion. In order to reach the multifaceted requirement of clinical bioadhesives, high adhesion strength, facile reversibility, and short curing time are all supposed to be achieved, which cannot be fully satisfied via a single adhesion mechanism.

#### Lubrication

The cornea, organ epithelium, and articular cartilage are natural paradigms of tribosystems where boundary lubrication plays a dominant role in the prevention of wear-induced damage [42]. Due to their surface property being compositionally analogous to natural tissues, the tribology of hydrogels has attained a lot of attention. Based on boundary lubrication, tribology in biofluids such as synovial fluid has been termed as hydration lubrication which allows for the influence of the hydration layer at the interface [43]. The normal coefficient of friction (COF) of hydrogel is within the range of 0.1–0.5 while the COF of natural articular cartilage and cornea is around 0.010 and 0.006 to 0.015, respectively [43–45]. So far, no artificial tribosystem has been developed to resemble the lubrication performance of human synovial joints under high local pressure (over 10 MPa) and a wide range of shear rates (from rest to 10^5^ to 10^6^ s^−1^). However, proper surface engineering has proven to efficiently enhance lubrication properties. For instance, pure hyaluronic acid (HA) is not capable of providing superior boundary lubrication while HA crosslinked with aggrecan exhibited much better lubrication performance as the lubricant with COF below 0.02 under 40 N load [46].

#### Cell affinity

Although hydrogels are alluring candidates for tissue engineering studies due to their compositional and mechanical similarity to natural tissues, cellular responses to the different hydrogels are diverse. Ligands, such as heparin, peptides, and aptamers are frequently used to enhance the cell affinity of hydrogels. The most famous affinity ligand is the arginine-glycine-aspartic acid (RGD) motif which is responsible for cell adhesion to the extracellular matrix (ECM), found in species ranging from Drosophila to humans. The RGD motif binds to the integrin on the cell membrane and can be chemically grafted on material surfaces to promote cell adhesion [47]. Biomedical hydrogels with cell affinity ligands enable a reliable hydrogel-tissue interaction and reduce the potential of an adverse host response.

#### Microstructure

The surface of natural organs exhibit diverse microstructures to enable geometry-derived functions. For instance, the ciliated columnar epithelium bears cilia on its apical surfaces to enable the transportation of mucus and the endothelial glycocalyx lining on the vascular endothelium acts as a mechanotransducer and barrier to blood flow. Apart from the extracellular matrix, the microstructure of the hydrogel surfaces also demonstrates the ability to provide a geometrically defined microenvironment to influence the cellular behavior. Nanosized rippled patterned and lotus leaf topographies generated on hydrogel surfaces by laser treatment have been reported to enhance hydrogel cell adhesion [48] while polyethylene glycol (PEG) hydrogels with surface microgrooves have demonstrated a width-dependent capability directing the migration of corneal epithelial cells [49].

#### Antimicrobial property

Microbial infections, caused by bacteria and fungi, pose a critical medical concern, especially with respect to surgical practice and wound dressing. With water constituting the majority, hydrogel also provides the ideal environment for microbial growth. The primary approaches to address this drawback include incorporating silver/gold nanoparticles, antibiotics, enzymes, and cationic polymers to generate a hydrogel-tissue interface that can eliminate the risk of infection [50]. Antimicrobial agents can be either simply added or chemically grafted into the network of hydrogels to ensure the sustained antimicrobial function mainly based on the bacterial membrane disruption mechanism.

#### Conductivity

The intrinsic ionic conductivity of the hydrogels (10^−5^ to 10^−1^ S·cm^−1^) is typically low compared to carbon-based (10^−1^ to 10^2^ S·cm^−1^) and metal-based electronic (e.g. copper ∼5.98 × 10^5^ S·cm^−1^, iron ∼1.04 × 10^7^ S·cm^−1^) [10,16,51]. Incorporating ions, carbon-derivatives, and conductive polymers are feasible methods to enhance the conductivity of hydrogels and the as-prepared hydrogels are defined as ionically or electrically conductive hydrogels. The increased hydrogel conductivity also helps decrease the impedance at the hydrogel-tissue interface which benefits the sensitivity of health monitoring and efficiency of electrical stimulation-based therapy [9,52–54]. Hence, the advancement of hydrogel-based bioelectronics is greatly affected by the surface electrical property of the hydrogel.

### Methods for hydrogel surface engineering

Surface engineering is the process of modifying the surface of a substrate to alter its properties. There are five basic strategies of surface engineering: (i) bottom-up and (ii) top-down modifications as well as (iii) mold-casting, (iv) interfacial assembly, and (v) wetting-enabled-transfer (WET) methods. Bottom-up surface engineering on hydrogels mainly refers to the *in-situ* polymerization techniques while top-down surface engineering on hydrogels mainly represents the deposition process of building blocks such as peptides, synthetic polymers, and biomacromolecules onto the hydrogel surfaces [55–57]. Mold-casting, interfacial assembly, and WET methods are mainly used to fabricate surface microstructures [58].

#### Surface chemistry engineering

**Figure 2.**
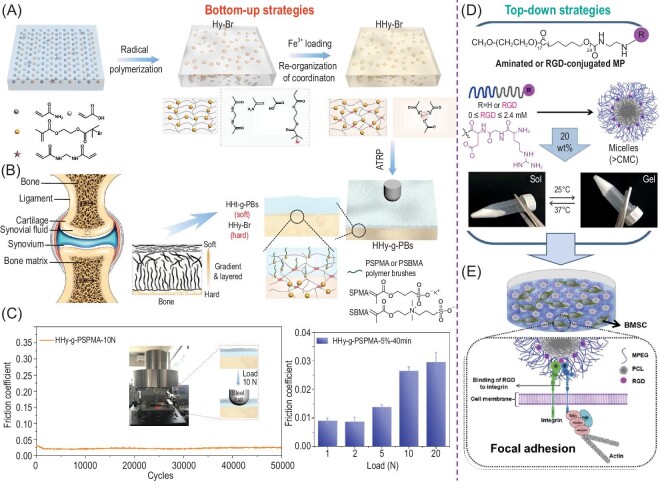
Surface chemistry engineering. (A) Schematics showing the synthesis route of PSPMA or PSBMA brush-grafted hydrogels, inspired by (B) natural articular cartilages. (C) The cycling friction coefficient of PSPMA-g-hydrogel under a normal load of 10 N at the frequency of 1 Hz and the friction coefficient of PSPMA-grafted hydrogel under different normal loads. (D) RGD modification of micelles for thermal-induced gelation. (E) Binding of RGD to integrin of cell membrane for bone marrow-derived mesenchymal stem cells (BMSCs) encapsulation. Adapted with permission from: (A–C) Ref. [60]. Copyright 2020 John Wiley and Sons. (D and E) Ref. [62]. Copyright 2020 Elsevier.

In biological systems, the hierarchically ordered structures are non-covalently assembled with continuous energy consumption. In order to resemble the hierarchical structure of biological systems, chemistry-based methods are frequently adopted for bottom-up surface chemistry engineering. The grafting of polymer brushes on substrate surfaces is considered as an effective strategy to tailor the interfacial chemical and physical characteristics. In this way, surface-initiated atom transfer radical polymerization (SI-ATRP) has been proposed by Zhou *et al.* as a versatile and efficient method for hydrogel surface functionalization [55,59–61]. The modification was realized by entangling polyelectrolyte brushes via *in-situ* polymerization at the subsurface of a stiff hydrogel substrate (Fig. 2A). The resulting hydrogel contains an outmost soft polymer layer on the stiffer hydrogel internal layer, mimicking the bilayer structure of natural articular cartilages (Fig. 2B) to enable hydration lubrication and wear reduction with low friction coefficients ∼0.020 (Fig 2C). Compared to the conventional hydrogel lubricant, the hydrophilic polyelectrolyte brushes-grafted hydrogels (i.e. poly(3-sulfopropyl methacrylate potassium), PSPMA) demonstrate the load-bearing capacity enabled by the relatively tough and elastic substrate hydrogel and lubrication property due to the surface-grafted PSPMA brushes. The friction coefficient increases from ∼0.009 to 0.030 with contact pressure rising from 3.97 MPa at 1 N to 10.8 MPa at 20 N which for the first time reaches the load at cartilaginous joints. Meanwhile, the hydrophilic polyelectrolyte brushes-grafted hydrogels (i.e. PSPMA) also demonstrate enhanced fouling resistance to a wide range of biological contaminants including proteins, enzymes, cells, and microbes, owing to the local hydration layer on hydrogel surfaces. In addition to the synthetic polymer brush-based modification, hydrogels with poor cell affinity and biocompatibility can be modified with RGD motif (Fig. 2D) to promote cell adhesion which unlocks their potential to serve as the bioactive porous scaffold for regeneration medicine (Fig. 2E) [62]. To summarize, the surface chemistry of a hydrogel can be modified by both bottom-up and top-down strategies.

#### Surface microstructure engineering

**Figure 3.**
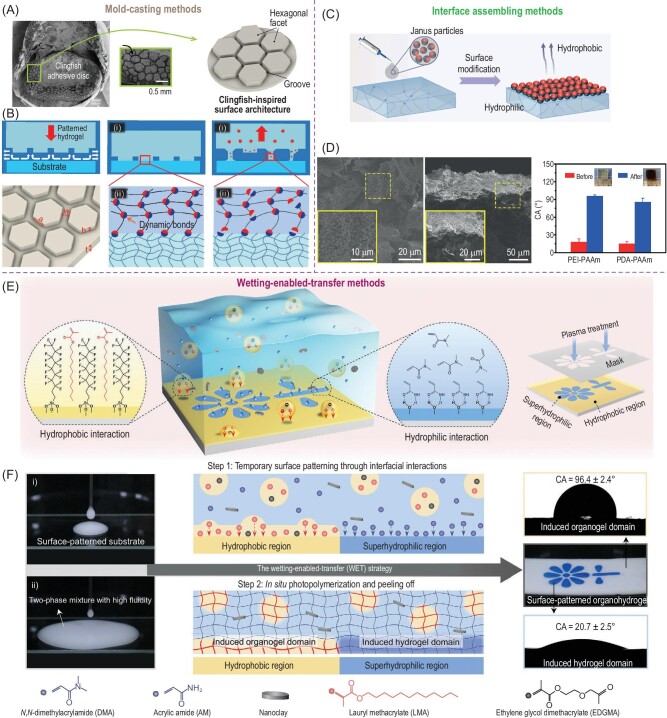
Surface microstructure engineering. (A) Clingfish-inspired adhesive surface architecture. (B) Schematic drawing of the microgrooves as water drainage channels to enable the rapid attachment of the hexagonal facets on the substrates while the tough connection enabled by dynamic bonds assists in energy dissipation to delay the detachment during separation. (C) Schematic drawing for the assembling of Janus silica particles on hydrogel surfaces with the (D) cross-sectional SEM images and contact angle on hydrogel surfaces before and after Janus silica particle decoration. (E) WET method for constructing discretionary surface patterns on organogel surfaces. The organogel monomers in the emulsions being actively absorbed on the hydrophobic regions of the pre-patterned substrate via hydrophobic interaction while the hydrogel monomers occupy the superhydrophilic regions of the pre-patterned substrate. (F) Through the WET strategy, the generated organogel and hydrogel domains exhibit the water contact CA ∼96.4 ± 2.4° and 20.7 ± 2.5°, respectively. Adapted with permission from: (A and B) Ref. [63]. Copyright 2018 John Wiley and Sons. (C and D) Ref. [66]. Copyright 2020 Elsevier. (E and F) Ref. [67]. Copyright 2021 John Wiley and Sons.

The surface microstructure of hydrogels refers to their topographical characteristics at the microscopic level and is commonly determined by their gelation process. Taking the adhesive hydrogel as an example, water entrapment inevitably occurs on hydrogels with a soft and flat surface and the thin water film confined at the hydrogel-tissue interface destroys the strong interfacial bonding and leads to an unstable connection. To solve this long-lasting obstacle, Gong *et al.* employed a silicone mold with honeycomb ridges to fabricate the polyampholyte hydrogel for underwater adhesion, mimicking the adhesive disc of the clingfish with hexagonal patterns and interconnected grooves (Fig. 3A) [63–65]. As shown in Fig. 3B, these interconnecting surface grooves act as channels to facilitate rapid water drainage during the underwater adhesion process which greatly promotes the tight contact and tough bonding between the adhesive hydrogel and the substrates underwater. In addition to the mold-casting methods, amphiphilic nanoparticle-based surface engineering was also proposed for fabricating hydrogels with surface microstructure. As shown in Fig. 3C, an oil-water or air-water interface is utilized to enable the interfacial assembly and generate the amphiphilic micro-silica particle-decorated hydrogel surfaces [66]. The resulting Janus silica microparticle (JSP) hydrogels not only exhibited an enhanced surface hydrophobicity for water retention due to the compactly assembled microparticle layer on the hydrogel surfaces (Fig. 3D) but are also capable of providing photothermal effects and photothermal antibacterial activities for therapeutic uses. In addition to the homogenous interfacial assembling, wettability manipulation-based printable surface modifications have been proposed recently to generate versatile surface patterns on the hydrogel surfaces. For instance, Wan *et al.* developed a printable surface patterning method, termed wetting-enabled-transfer (WET) method, on organohydrogel surfaces (Fig. 3E) using two immiscible liquids (i.e. organogel/hydrogel precursor solution) [67]. A prepatterned substrate with hydrophobic/hydrophilic domains was used to induce the differentiated attachment of oil-in-water emulsions based on the wettability match followed by photocuring to immobilize the corresponding microstructure (Fig. 3F). The WET strategy demonstrates its connivence in surface patterning on organohydrogel surfaces without the use of high-end apparatuses and expensive molds.

## ENGINEERING ASYMMETRIC SURFACE FUNCTIONS ON HYDROGELS

In this section, the desirable surface functions of Janus hydrogels at their opposite sides in clinical procedures are first discussed. Afterward, particular consideration is paid to the methods for engineering both the integrally constructed or post-assembled Janus hydrogels.

### Basal surface for hydrogel tissue integration

The basal side of Janus hydrogels applied on soft tissues generates a new interface where interfacial bonding dominates the hydrogel-tissue integration. For advancing the performance of bioadhesives, two categories of interactions are considered to induce surface adhesion: non-covalent interactions for instant attachment and covalent interactions for tough bonding with the target tissues. Synergizing the advantages of both covalent and non-covalent interactions for conquering the weakening effect from surface hydrations on tissues remains challenging but pivotal for the development of the next generation of wound dressing and tissue adhesives. In this section, the strategies for realizing instant and robust tissue adhesion are comprehensively discussed which can be summarized into two categories: surface water-repelling and surface water-removing strategies.

#### Surface water-repelling strategy

**Figure 4.**
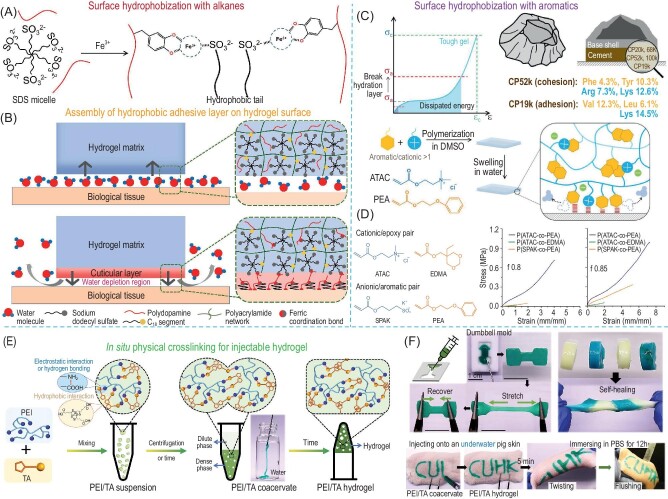
Schematic illustration of (A) Fe^3+^-induced reconstruction of SDS micelle and (B) assembly of a soft armour-like hydrophobic adhesive layer over the prehydrogel. (C) The cationic and hydrophobic amino acid contents in the two cement proteins of barnacle. Design principle of strong adhesive hydrogel of poly(ATAC-co-PEA) with π–π and cation–π interactions contributing to the dynamic cohesive strength and dehydration at the interface to promote underwater adhesion. (D) Effect of different hydrophobic monomers (monomer fraction of f = 0.8 and f = 0.85) on mechanical strength of the hydrogel. (E) Schematic illustration of the formation of coacervate-based polyethylene imine and thioctic acid (PEI/TA) hydrogel. (F) Injection of PEI/TA coacervate on glass plate and porcine skin immersed in water and maintaining stable attachment after 12 h. Adapted with permission from: (A and B) Ref. [28]. Copyright 2022 Elsevier. (C and D) Ref. [69]. Copyright 2018 John Wiley and Sons. (E and F) Ref. [73]. Copyright 2022 John Wiley and Sons.

One emerging strategy is to modulate the surface hydrophobicity of hydrogels based on water-repelling mechanisms. For instance, Pan *et al.* reported the development of a hydrophobic catechol-containing hydrogel surface by the Fe^3+^-induced recombination of alkyl moieties belonging to the micelle crosslinking network (Fig. 4A) [28]. The obtained hydrophobic adhesive surface resembles the protective cuticle structure on Mytilus byssal thread and exhibits an instant anchoring to the substrates that are submerged in aqueous media (Fig. 4B). The hydrophobic surfaces of the adhesive hydrogel generate a water depletion region to shield the adhesive function of catechol groups on the substrates. Meanwhile, Fan *et al.* reported a synthetic cation-aromatic adhesive system inspired by the reversible interaction chemistry of barnacle cement proteins (Fig. 4C) [68,69]. The aromatic moieties of the hydrogel not only contribute to the surface hydrophobicity-induced water-repelling functions during adhesion formation but also form the cation-π interactions with the adjacent quaternary ammonium groups for energy dissipation at the near interface region. From the results of the control groups (Fig. 4D), the tensile strength of the hydrogels prepared by the cation-aromatic pair is 3- to 10-fold greater than the cationic/epoxy and anionic/aromatic pairs. This type of adhesive system is most effective on the negatively charged substrates which are also very common substrates in our lives including most of the tissues, glass, and plastics. Besides the patch-like bulk hydrogels, physically crosslinked hydrogels exhibit both fluidity under shear stress (shear-thinning) and time-dependent re-crosslinking upon relaxation, which have been widely used as the injectable hydrogels delivered to the target area by a syringe [70,71]. The underwater adhesion of these injectable hydrogels can be divided into 2 steps: (1) during the injection of these viscous hydrogel flows on the wet substrate, the surface hydration is replaced by the hydrogels which induces a conformal hydrogel-substrate contact; (2) after injection, the polymer network of the hydrogel reconstructs to strengthen the hydrogel adhesion with the assistance of both the interfacial interaction and topological entanglement [72]. For example, Peng *et al.* reported a coacervate-derived hydrogel using polyethylene imine and thioctic acid (PEI/TA), which can effectively repel interfacial water and fill up the substrate surface irregularities, thereby forming tight contact with the substrates and achieving robust and instant adhesion on various wet and underwater substrates (Fig. 4E) [73]. Compared to many of the previously reported injectable hydrogels that need complicated external stimuli for post-crosslinking, the spontaneous aggregation of hydrophobic 1,2-dithiolanes of TA molecules induces their cross-linking with PEI and enhances the cohesion of the coacervate, resulting in the coacervate–hydrogel transition *in situ* (gelatin time ∼120s) without any additional stimuli. The physically crosslinked PEI/TA hydrogel is highly modulable and can be directly applied on wet substrates as the underwater adhesives and sealants for a wide range of surfaces such as glass, metal, rubber, Teflon, wood, and tissue (Fig. 4F).

#### Surface water-removing strategy

**Figure 5.**
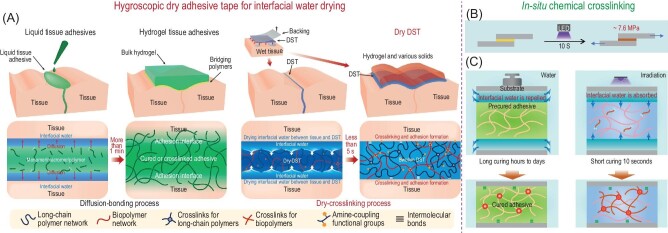
(A) Comparison of liquid tissue adhesives and dry double-side adhesive tape (DST) with the proposed interfacial water removing mechanism. (B) Schematic illustration of underwater adhesives based on an *in situ* photocuring strategy. (C) Comparison of two underwater adhesion strategies using precured adhesive and precursor gel. Adapted with permission from: (A) Ref. [74]. Copyright 2019 Springer Nature. (B and C) Ref. [77]. Copyright 2021 American Chemical Society.

Contrary to the water-repelling strategy, the body liquid-removing strategy based on hygroscopicity is also effective on bulk adhesives. Inspired by the function of adhesive tape used for mounting objects on dry substrates, Yuk *et al.* proposed a dry double-sided tape prepared by a biopolymer (gelatin or chitosan) and crosslinked poly(acrylic acid) (PAA) grafted with N-hydrosuccinimide (NHS) ester for adhesion on wet tissues [74–76]. The negatively charged carboxylic acid groups of the PAA facilitate instant adhesion by quick hydration to remove the interfacial water and form hydrogen bonds with the tissue surfaces (Fig. 5A). After that, the NHS-ester grafted on PAA interlinks with the amine groups of the tissues by amide crosslinking to strengthen the adhesion. The adhesive tape is stretchable to ∼17 times of its original length and can form a stable attachment to the beating heart within 5s and maintain its integrity and adhesion after 3 days. Compared to the clinical suture mounting that is time-consuming and requires complicated operations, this hydrogel-based adhesive tape demonstrated its advantage of increased mounting efficiency and simplified operations on dynamic tissues. Meanwhile, chemical crosslinking-induced solidification also demonstrates its unique characteristics in wet adhesion. To address the drawbacks of conventional chemical crosslinking methods that are time-consuming, namely, require a long curing time. Recently, Wang *et al.* proposed an *in situ* photocuring strategy for developing underwater adhesives [77]. Under ultraviolet (UV) irradiation for 10s, the precursor solution containing water-soluble photoinitiator, acrylic acid, and chemical crosslinker rapidly solidifies to a bulk adhesive with the adhesion strength of up to 7.6 MPa on glass substrates (Fig. 5B). Compared to the conventional pre-cured adhesives that require a long adhesion time to repel the surface hydration and generate surface bonding, the hygroscopicity precursor solution adsorbs the interfacial water and rapidly solidifies upon *in situ* photocuring to facilitate the construction of strong adhesive–adherend interactions. The selection of the photoinitiator is also vital to this *in situ* chemical crosslinking strategy (Fig. 5C). The extinction coefficient of modified initiator diphenyl (2,4,6-trimethylbenzoyl) phosphine oxide used in this work is over 10-fold higher than common initiator I2959 and camphorquinone which contributes to the fast-curing process of the precursor solution, which promotes the use of liquid adhesives in broad underwater conditions.

### Apical surface as temporary biological barriers

Most existing hydrogel adhesives have symmetric surface adhesion functions which enable stable adhesion on the side facing the tissue surfaces but fail to serve as the temporary barriers substituting the epidermis and endothelium. In most *in vivo* applications, single-side bioadhesives are preferred with specific surface function design at the basal side facing body fluids and apical side as the temporary biological barrier replacing the original ones.

**Figure 6.**
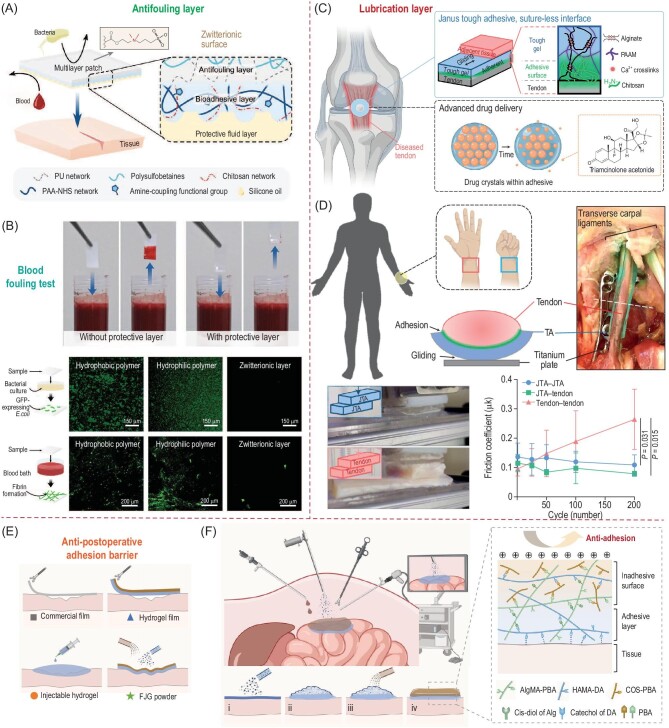
(A) Schematics of a multilayer patch comprising the bioadhesive layer interconnected with a hydrophobic fluid layer on the adhesive side and an antifouling polymer layer on the non-adhesive side. (B) Blood fouling tests of multilayer patches with and without the antifouling layer; fluorescence images showing the antimicrobial property of the multilayer patches with *E. coli*. (C) Schematics showing a Janus tough adhesive (JTA) that consists of a tough hydrogel dissipative matrix and a single side chitosan-derived adhesive surface for dry delivery. (D) Images of the JTA gliding with surrounding tissues (i.e. volar plates and transverse carpal ligaments) in human cadaveric wrists during extension and flexion; frictional properties of JTA with a cyclic loading of 8 N. (E) Illustration of fast-Janus-gelation (FJG) powder compared to other types of antiadhesion barriers. (F) Schematics showing the application of FJG powder in endoscopic surgery; (i–ii) delivery of the FJG powder to the tissue surface that undergoes gelation with tissue surface hydration; (iii) hydration of FJG powder with surface moisture to form an adhesive hydrogel layer. (iii–iv) Spraying cationic solution on the upper unhydrated surface to form the anti-adhesion barrier; bottom right diagram showing the details of the Janus hydrogel network. Adapted with permission from: (A and B) Ref. [1]. Copyright 2021 John Wiley and Sons. (C and D) Ref. [78]. Copyright 2022 Springer Nature. (E and F) Ref. [79]. Copyright 2023 National Academy of Sciences.

The opposing layer of the bioadhesives is typically designed to prevent the potential postoperative risks and host responses in the presence of body fluids such as blood, mucus, and microbials. To address these issues, Wu *et al.* proposed a novel strategy for a minimally invasive tissue sealant based on a multilayer bioadhesive patch with a hydrophobic fluid layer to repel interfacial water, a bioadhesives layer, and an antifouling zwitterionic non-adhesive layer (Fig. 6A) [1]. The non-adhesive layer is prepared using a zwitterionic-interpenetrated elastomer to resist foulant adsorption by its hydration shell in aqueous media. As shown in Fig. 6B, the protective antifouling layer enables a great resistance to blood fouling, bacterial adhesion, and fibrinogen adsorption, which also reduces the risk of inflammation on the adhesive-attached area and improves long-term stability. For loading-bearing materials such as tendons and ligaments that actively stretch and contract, their friction force with surrounding tissues should be relatively low. Freedman *et al.* reported a Janus tough adhesive (JTA) with robust adhesion versus tissues and gliding properties on opposing surfaces to serve as a high-capacity drug depot and delivery system for tendon healing (Fig. 6C) [78]. The model drug corticosteroid, triamcinolone acetonide (CORT) is loaded in the hydrogel as the microcrystal with the concentration 25 000× its solubility limit, which enables a sustained release of CORT to reduce inflammation, modulate chemokine secretion, recruit tendon stem and progenitor cells, and promote macrophage polarization to the M2 phenotype. Meanwhile, the low coefficient of friction of the opposing surface of JTA with surrounding tissues helps reduce fibrotic scar formation and protects tendon injury following bone fracture fixation (Fig. 6D).

The apical non-adhesive layer of bioadhesives also helps prevent postoperative fibrosis. For instance, Jia *et al.* developed sprayable fast-Janus-gelation (FJG) powder for minimally invasive surgery (Fig. 6E) [79]. Upon delivery on wet tissue surfaces, FJG powder quickly hydrates to form an adhesive hydrogel layer on the tissue surface while a positively charged solution is subsequently sprayed on the apical powder to neutralize the adhesive capability of catechol and carboxyl moieties. The as-developed Janus-adhesive hydrogel barrier effectively reduces the abnormal fibrotic connection between the target organ and surrounding tissues which is promising as a temporary barrier to prevent postoperative adhesion (Fig. 6F).

### Methods for engineering Janus hydrogels

**Figure 7.**
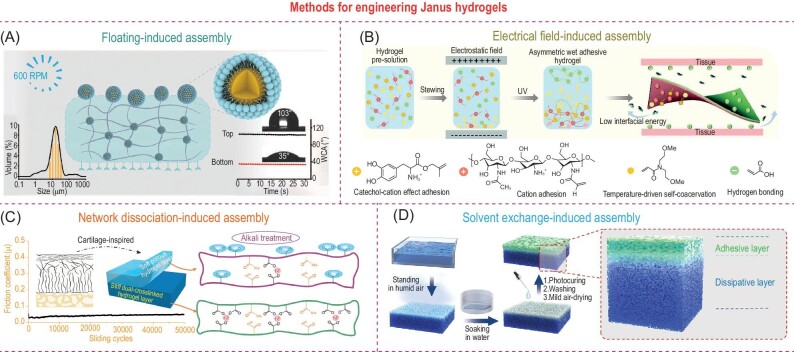
Methods for engineering Janus hydrogels. (A) The formation of asymmetric structure driven by emulsion floatation (left inset is particle size) at a stirring speed of 600 r/min with the water contact angle measurement at the top/bottom surfaces (right inset). (B) Molecular design of asymmetric adhesion hydrogels under an electrical field. (C) Formation mechanism of the soft hydrogel layer by the alkali-induced network dissociation with load-bearing long-term lubrication property. (D) The manufacturing procedures include moisture-induced phase separation, solvent exchange, photocuring, and washing to generate the adhesive and dissipative layers. Adapted with permission from: (A) Ref. [24]. Copyright 2023 John Wiley and Sons. (B) Ref. [80]. Copyright 2023 John Wiley and Sons. (C) Ref. [81]. Copyright 2020 American Chemical Society. (D) Ref. [82]. Copyright 2024 John Wiley and Sons.

Asymmetric surface functions on hydrogels can be achieved through integral construction or post-assembly methods. External forces (e.g. buoyancy and electrical forces) are effective tools for integrally generating heterogeneous structures. The size-dependent flotation phenomenon of emulsions was proposed by Wang *et al.* to generate asymmetric crosslinking during the curing process of the hydrogels (Fig. 7A) [24]. By controlling stirring speed, emulsion droplets float to occupy the hydrogel apical surface and hinder the adhesive groups while the functional groups at the bottom surface were fully exposed to enable the robust hydrogel-tissue connection. The difference in the adhesive property at the opposing surfaces was modulated by the interfacial hindrance of emulsions, thus reducing apical adhesion-induced postoperative adhesion. Similarly, Xu *et al.* reported the use of an electrical field to facilitate the distribution of cationic and anionic monomers in asymmetric hydrogel during UV curing (Fig. 7B) [80]. The basal surfaces with cationic catechol moieties generate the underwater adhesion property while the top surfaces with anionic acid moieties exhibited a relatively low tissue adhesion. Besides the integrally formed ones, asymmetric hydrogels can also be engineered by post-modification methods. For instance, in order to imitate the layered structure of the articular cartilage and reproduce its load-bearing lubrication performance, Qu *et al.* introduced an alkali-induced network dissociation strategy to assemble a soft and porous layer on the top of a tough double-layer hydrogel (Fig. 7C) [81]. Hence, the combination of the top loose layer for lubrication and the bottom dense layer for energy dissipation ensures outstanding wear resistance features, demonstrating a coefficient of friction lower than natural articular cartilage under high load. The solvent exchange method is another efficient approach for preparing heterogeneous structures. Very recently, Ma *et al.* reported the use of hygroscopicity of dimethyl sulfoxide (DMSO) that actively absorbs vapor from air and generates a layer of porous tough apical polyvinyl alcohol (PVA) network at the air-gel interface (Fig. 7D) [21,82]. Afterwards, the precursor containing N-hydroxysuccinimide ester and trimethylammonium moieties is introduced on this porous apical surface to obtain the adhesive layer. The electrostatic crosslinking within the adhesive layer and high crystallinity of the PVA dissipative layer reduces the swelling ratio, while the asymmetric adhesion properties prevent the occurrence of postoperative adhesion during tissue repair. Other post-assembly strategies such as 3D printing and *in-situ* multi-layer spraying-photocuring methods are summarized in Table 1, along with their potential for clinical translation, comprehensively discussed below.

**Table 1.** List of Janus hydrogels with their preparation strategies and potential clinical applications.

Janus structure	Strategy	Clinical applications	Ref.
Gradient porous network with asymmetric micelle distribution	Flotation-induced assembly	Singe side adhesive tissue sealant, strain sensor	[24]
Negatively charged apical layer; positively charged basal layer	Electrostatic field-induced assembly	Singe side adhesive tissue sealant	[80]
Soft porous apical layer; dense stiff basal layer	Alkali-induced network dissociation	Artificial cartilage	[81]
Dense and smooth basal layer; porous apical layer entanglement with adhesive polymers	Moisture-induced phase separation, solvent exchange	Singe side adhesive tissue sealant	[82]
Neutral charged apical layer; negatively charged basal layer	Endoscopic batch delivery, hygroscopic gelation	Minimally invasive organ repair surgery, anti-adhesion barrier	[79]
Sticky dry hydrogel bottom layer; zwitterionic antifouling top layer	Layer-by-layer construction process	Hemostasis; tissue sealing patch	[83]
Asymmetric adhesion	Sol-adhesive to gel-nonadhesive process	GATA6^+^ cavity macrophage trap	[84]
Gradient-arranged hydrophilic fractal microchannels	Flotation-induced assembly	Burn wound healing	[86]
Asymmetric adhesion	Sol-adhesive and gel-nonadhesive processes	Cardiac regeneration	[89]
Two hydrogel layers encapsulating different therapeutic agents	3D printing	Bacterial elimination and angiogenesis for wound healing	[90]

## POTENTIAL OF JANUS HYDROGELS FOR CLINICAL TRANSLATION

By orchestrating surface structures and functions, Janus hydrogels offer great opportunities for clinical translation. In this section, Janus hydrogels successfully developed for theranostic applications regarding hemostasis, tissue sealant, chronic wound management, and regenerative medicine are comprehensively discussed with their potential to improve patient outcomes.

### Hemostasis and tissue sealant

**Figure 8.**
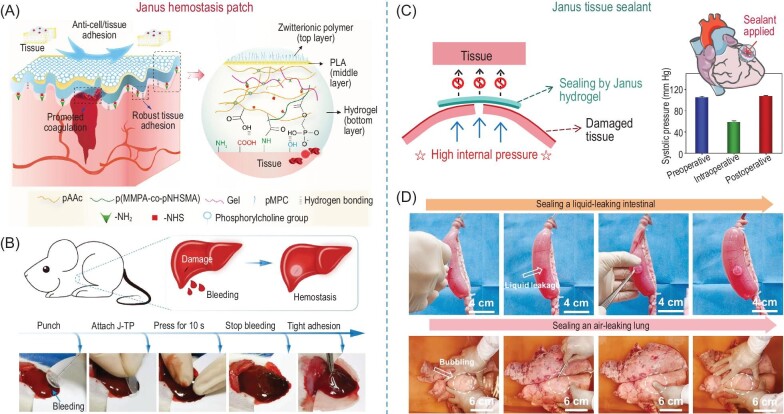
(A) Schematic illustration of the design and structure of Janus tissue patch. (B) Demonstration of Janus tissue patch applied on a bleeding liver of a rat with procoagulation property. (C) Janus tissue patch for penetrating wound on porcine heart wall with measured arterial blood pressure. (D) The sealing performance of Janus tissue patch on a liquid-leaking intestinal and an air-leaking lung. Adapted with permission from: (A and B) Ref. [83]. Copyright 2023 John Wiley and Sons. (C and D) Ref. [82]. Copyright 2024 John Wiley and Sons.

Bioadhesive hydrogels hold tremendous potential in controlling and managing bleeding in cases of tissue damage and surgical wounds. Hydrogels designed for hemostasis should bear the capability of instant underwater adhesion and procoagulant function. A tight attachment is the initial step when the hydrogel is applied to the bleeding wound area, followed by the accelerated coagulation process induced by the hydrogel-blood interaction. In order to simultaneously enable an efficient wound closure and inhibit postoperative tissue adhesion, Peng *et al.* developed a Janus tissue patch with an antifouling zwitterion-grafted apical layer, a thin polylactic acid (PLA) skeleton middle layer, and a bioadhesive bottom layer (Fig. 8A) [83]. Instant tissue adhesion is enabled by the water-removing mechanism of the poly acrylic acid moieties and robust bonding is generated between N-hydroxy succinimide (NHS) groups from the hydrogel side and amine groups from the tissue side. The elevated hemostasis performance is ascribed to the rapid water adsorption capability of the hydrogel bottom layer that concentrates the coagulation factors and abundant phosphates exposed to the wound area as the procoagulant that activates coagulation factors XI and XII (Fig. 8B). Meanwhile, the antifouling performance is also verified on the zwitterion (phosphorylcholine)-grafted apical layer, demonstrating great resistance to *Staphylococcus aureus* and L929 cells. Such an asymmetric surface function design well meets the surgical demands for advanced internal wound healing. In addition to the hemostasis, the sealing performance is also important for repairing the organ rupture. Specifically, the fluid leaking from the aorta, stomach, and intestine ruptures and the air leaking from a lung lobe rupture raises higher requirements for adhesives to resist the internal pressure at the damaged area. For instance, a Janus tissue patch was reported to treat the penetrated wound on a porcine heart wall. The hemostasis was achieved by pressing the patch on the wound area within 30 s with the arterial blood pressure recovering from a life-threatening situation (59/43 mmHg) to the normal range (107/82 mmHg) (Fig. 8C) [82]. The burst pressure on the porcine skin of another reported tissue patch was measured to be 493 mmHg which is much higher than the internal pressure of humans including air pressure of the lung lobe and gastrointestinal fluid pressure (Fig. 8D) [24]. The outstanding sealing performance also prevents the postoperative re-leaks of wounds under continuous contraction and extension of organs.

### Chronic wound management

**Figure 9.**
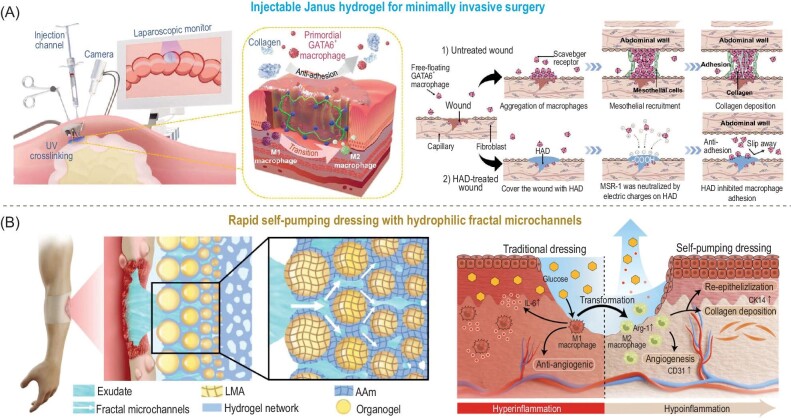
(A) Schematics showing photocurable catechol-grafted hyaluronic acid (HAD) for injection via laparoscope with the mechanism for preventing GATA6^+^ macrophage-induced abdominal adhesion. (B) Schematics showing a self-pumping Janus organohydrogel with rapid burn wound exudate drainage and its mechanisms for accelerating the healing process. Adapted with permission from: (A) Ref. [84]. Copyright 2021 John Wiley and Sons. (B) Refs [86,87]. Copyright 2023/2024 John Wiley and Sons.

Chronic wounds often refer to wounds that stay in the inflammatory stage for weeks or years. Hydrogels are widely reported for wound management with pro-healing properties which are realized by a variety of mechanisms such as providing a moist and weak acid environment, protective barrier effect against microbes/contaminants, sustained release of drugs, and elimination of reactive oxygen species (ROS) [22]. For internal chronic wounds, the injectable Janus hydrogel with apical non-adhesive polyanion surfaces was reported to neutralize scavenger receptors, thereby inhibiting collagen deposition and uncontrolled recruitment of the GATA-binding factor 6 (GATA6^+^) cavity macrophages (Fig. 9A) [84]. Meanwhile, the barrier effect and stable tissue retention of the hyaluronic acid-based apical layer promotes the transition of pro-inflammatory M1 phenotype to anti-inflammatory M2 macrophages, contributing to tissue regeneration. Another challenging area of wound management is the treatment of burn wounds from which the swollen tissue secretes excessive exudates, delaying the healing process. A Janus hydrogel dressing with rapid self-pumping was accordingly proposed and developed to enable the fast exudate-drainage on burn wounds [85]. Very recently, Lan *et al.* reported the development of fractal hydrogel microchannels via a dynamic floating-colliding-coalescing process within the wound dressing (Fig. 9B), demonstrating a draining efficiency ∼30 times faster than the conventional homogeneous hydrogel [86]. The unidirectional drainage process is driven by the Young-Laplace pressure force within the fractal microchannels from the basal to the apical layer. The fast exudate-removal process ensures a pro-healing environment for burn wounds, hence, reducing dermal cavity, inhibiting inflammatory response, accelerating angiogenesis, and promoting hair follicle regeneration [87].

### Regenerative medicine

**Figure 10.**
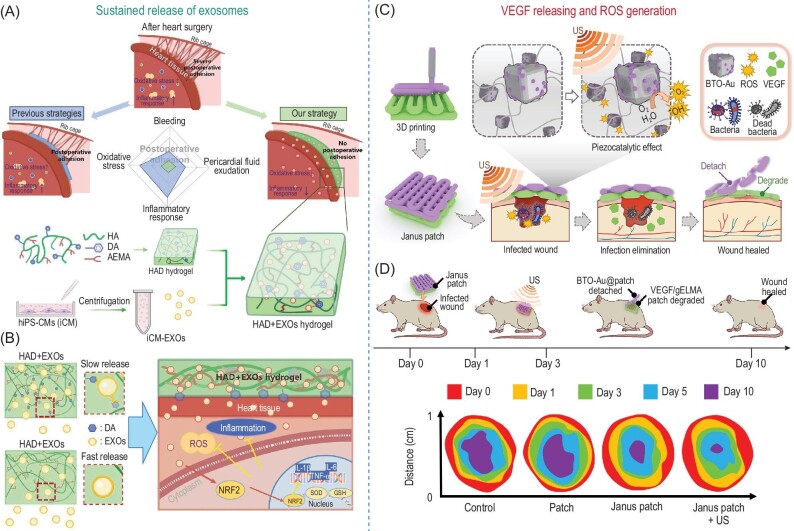
(A) Illustration of *in-situ* photocurable hydrogels loaded with stem cell–derived cardiomyocyte exosomes (iCM-EXOs) to avoid stop bleeding, exudating pericardial fluid, reduce oxidative stress, and inhibit inflammatory response related to postoperative pericardial adhesion. (B) The use of catechol groups for the sustained release of iCM-EXOs that downregulates oxidative stress and inflammation. (C) Schematics showing the fabrication of the Janus hydrogel patch with piezoelectric gold-nanoparticle-decorated tetragonal barium titanates (BTO-Au) for ultrasonic microbial elimination and VEGF for angiogenesis. (D) Illustration of the application of the Janus patch and sonodynamic antibacterial treatment at the early stage of wound healing; size of wound area during the healing process with different treatments. Adapted with permission from: (A) Ref. [93]. Copyright 2023 AAAS. (B) Ref. [94]. Copyright 2023 AAAS.

Tissue and organ damage or dysfunction resulting from trauma, disease, and aging are persistent issues in the fields of biomedicine and healthcare. Injectable or implantable hydrogels are identical porous scaffolds for delivering therapeutic agents (i.e. drugs, proteins, and cells) to actively promote tissue regeneration [88]. Hydrogel-based regenerative medicine, especially those with delicate surface and structure design is of growing interest. In order to accelerate myocardial recovery after heart surgery, Wang *et al.* reported the development of a pluripotent stem cell-derived cardiomyocyte exosomes (iCM-EXOs)-encapsulated Janus hydrogel with asymmetric adhesion inhibiting fibrous connections to the chest cavity (Fig. 10A) [89]. Meanwhile, the catechol-assisted sustained release of iCM-EXOs (Fig. 10B) alleviates oxidative stress and improves cardiac function. The apical layer with polyanionic HA inhibits the recruitment of macrophages from the thoracic cavity, thus preventing the GATA6^+^ cavity macrophage clearance of iCM-EXOs. The non-adhesive apical layer not only acts as a physical barrier to resist macrophage accumulation but also ensures the long-term pharmacological effects of iCM-EXOs loaded within the hydrogel. In addition to the delivery of exosomes, the Janus hydrogels also functionalized as the reservoir to enhance the therapeutic effect of nanomaterials. In order to avoid the undesirable clearance of nanomaterials, Huang *et al.* developed a Janus piezoelectric hydrogel patch via 3-dimensional printing for sonodynamic bacteria elimination and wound healing (Fig. 10C) [90]. The gold-nanoparticle-decorated tetragonal barium titanates (BTO-Au) were immobilized in the apical layer which produced ROS to eliminate pathogens under ultrasound irradiation. The vascular endothelial growth factor (VEGF) was loaded within the basal layer prepared by bioabsorbable methacrylate gelatin (GelMA). The use of BTO-Au for ROS generation avoids the antibiotic resistance and undesirable exposure of nanomaterials to blood circulation that may cause potential adverse effects. When used at a microbe-infected wound, the ultrasound irradiation was applied to cover the inflammation stage for infection elimination (Fig. 10D). Afterwards, the BTO-Au-loaded apical layer was removed, leaving the VEGF-loaded basal layer promoting cell adhesion and angiogenesis. The bioabsorbability of the GelMA basal layer avoids the removal of patches. Similar to the cocktail treatment, the combination therapy from both the VEGF releasing from the basal layer and ROS from the apical layer demonstrated a significant acceleration in the wound healing process with personalized and programmable features for commercialization.

These Janus hydrogels, which possess a unique structure with two distinct sides, have the potential to address various medical challenges. Several innovative paradigms have been demonstrated for designing and fabricating Janus hydrogels, including their use in health monitoring, hemostasis, wound healing, electrical or mechanical stimulation therapy, and regenerative medicine. Compared to conventional hydrogels with symmetric surface functions, Janus hydrogels are developed to overcome the challenges associated with clinical translations.

## CONCLUSIONS, CHALLENGES, AND FUTURE PERSPECTIVES

Bioadhesive hydrogels have rapidly emerged as a dynamic biomaterial, merging multidisciplinary fields such as material physics/chemistry, surface science, and biology. This paper aims to provide an overview of the current progress on tailoring the asymmetric surface functionalities of hydrogels, specifically, Janus hydrogels, for biomedical applications. The basic concept of hydrogel surface functionalities and methods for surface chemistry and geometry engineering were first discussed. These methods are applied to develop Janus hydrogels with distinct surface functionalities, broadening their practicality as a bioadhesive interface in complex physiological environments for a range of clinical procedures.

In detail, the basal side of Janus hydrogels, facing biological tissues, facilitates instant (several seconds to ∼5 min) and robust wet adhesion (2- to 5-fold higher than commercial tissue glue), overcoming the weakening effect from surface hydration on wet tissues. Efficient strategies include *in situ* physical crosslinking or surface hydrophobicity-induced water-repelling and *in situ* chemical crosslinking or surface hygroscopicity-induced water-removing. Meanwhile, the opposing side of Janus hydrogels acts as a temporary barrier facing the surrounding tissues which actively reduces postoperative adhesion with other tissues, resists undesirable protein deposition and corresponding inflammation, and prevents postoperative scar formation via their lubricative property. These designs enhance the viability of Janus hydrogels as the tissue sealant replacing sutures and staples for wound closure, dressings for chronic wound management, and a platform for delivering therapeutic agents.

Although impressive progress has been achieved in Janus hydrogels as the bioactive interfaces, some critical issues and technical challenges remain to be explored. (1) Most currently reported Janus hydrogels were characterized in the normal body fluids (e.g. PBS and sweat). Patients with metabolic syndrome of diabetes, high blood pressure (hypertension), and obesity have diverse internal physiological conditions. More fundamental research should be devoted to the effect of complicated biofluids on the performance of apical-basal hydrogel functions. (2) Bioabsorbable Janus hydrogels as temporary soft implants have demonstrated their convenience in preventing second surgery for hydrogel removal. Research regarding the *in-situ* degradation of the implanted hydrogels at the wound sites is limited. Considering the complexity of the tissue-specific healing process and the involvement of pro-healing agents, can we precisely tun the degradation rates according to the microenvironment to match the hydrogel degradation rate and wound healing rate? (3) The integration of living cells and nonliving polymer matrices holds great promise in clinical applications such as artificial tissues or regenerative medicine. However, the immune-induced clearance of the allogeneic cells remains a critical issue. How can we design the Janus hydrogel from both the surface chemistry and microstructure aspects to enable selective permeation for prolonged cell survival and their sustained secretion of therapeutic extracellular vesicles?

Janus hydrogels, as a new type of artificial tissue-interactive interface, are still in their infancy. While many innovative methods have been reported to construct Janus hydrogels mimicking natural biological barriers, their clinical translation requires multidisciplinary convergence. For example, combining technologies such as synthetic biology, and selective recruitment of epidermal/endothelial cells instead of immune cells on the apical layer of Janus hydrogels could facilitate host response-free implantation. With the improved understanding of physiological barriers, we believe that the dream of Janus hydrogels being widely used as soft implants in medicine will become a reality.
